# Dataset for the electronic customer relationship management based on S-O-R model in electronic commerce

**DOI:** 10.1016/j.dib.2022.108039

**Published:** 2022-03-12

**Authors:** Bui Thanh Khoa

**Affiliations:** Industrial University of Ho Chi Minh City, Ho Chi Minh City, Viet Nam

**Keywords:** Electronic loyalty, Perceived mental benefits, Hedonic value, Electronic commerce (e-commerce), Electronic customer relationship management (e-CRM)

## Abstract

Customer loyalty is difficult to establish because of the danger of online transactions, which causes risk in all transaction procedures. The dataset presents the survey data including three factors as electronic loyalty, perceived mental benefits, hedonic value. The quantitative data is based on 485 participants who bought from e-commerce websites. SmartPLS 3.7 software analyzed the survey collected data in three stages: measurement model evaluation (scale reliability and scale validity); structural model assessment (collinearity issues, the significance and relevance of the structural model relationships, coefficient of determination, effect size, and predictive relevance); and mediator analysis. Aside from confirming the Stimulus – Organism - Response (SOR) model in the relationships between perceived mental benefits, hedonic value, and electronic loyalty; moreover, this data revealed that hedonic value had a mediating effect on the relationship between electronic loyalty and perceived mental benefits in the electronic customer relationship management.

## Specifications Table

**Table:** 

Subject	Marketing, Applied Psychology
Specific subject area	Electronic commerce, online shopping, Stimulus – Organism - Response (SOR) model, consumer behavior
Type of data	Survey data, tables, and figures
How the data were acquired	The dataset was created by collecting data through an online questionnaire in Google Form. The major scale of constructs is adopted from prior studies. The questionnaire was disseminated through social media to respondents; the scale's content is included as a supplementary file.
Data format	Raw data and analyzed statistical dataset.
Description of data collection	Data for quantitative analysis was gathered via an online self-administered questionnaire to customers who frequently purchase online, such as student, white-collar employees, business owners, lecturers, housewives, workers, and government officials. The convenience sampling technique was used to select the survey data. The Google Form link was distributed through social media platforms like Facebook and Zalo. The sample size for this research was 485 participants in the three most prominent cities in Vietnam.
Data source location	City: Ha Noi city, Da Nang city, Ho Chi Minh city Country: Vietnam
Data accessibility	Khoa, Bui Thanh (2021), “Dataset for the electronic customer relationship management based on S-O-R model in electronic commerce,” Mendeley Data, https://data.mendeley.com/datasets/n9tdpdp45k Repository name: Mendeley Data Link: https://data.mendeley.com/datasets/n9tdpdp45k
Related research article	None

## Value of the Data

• This data set supplies the survey data to understand customer perception as perceived mental benefits, hedonic value, and electronic loyalty in the online environment.
• These data have a beneficial impact on theory and practice, helping researchers and businesses that wish to learn about consumer insights.
• Researchers can use this data to extend relationships in a model such as the moderating effect of hedonic value on the relationship between perceived mental benefits and electronic loyalty.
• Additionally, these data have shown a positive connection between the study constructs; therefore, consultants may depend on the information to propose appropriate governance implications for online companies.
• Finally, this data set may be run by chi-square, independent sample T-test, and analysis of variance (ANOVA) on groups such as gender, occupation, and online shopping times per month.

## 1 Data Description

Domestic and foreign major companies invested billions in Vietnam's e-commerce industry. In 2018, five Vietnamese companies were among Southeast Asia's top-ten most-visited e-commerce websites [1]. The ecosystem has seen rapid development in terms of product diversity, technical infrastructure, and support services. E-commerce models for consumer-to-consumer, business-to-business, and consumer-to-business transactions have developed gradually over time [2]. Emerging business ideas include selling through social media and mobile phones [3]. The evolution of selling from websites to live broadcasts demonstrates the worldwide e-commerce industry's growth. Electronic businesses may potentially benefit from the growing digital content industry. Additionally, many domestic and foreign businesses have begun to develop cross-border e-commerce. This market will continue to expand in the coming years [4].

The stimulus–organism–response (SOR) model, developed by Mehrabian and Russell [5], is a well-known paradigm for describing buyer–seller interaction and is widely used in consumer behavior research [6]. Consumers who purchase a product or service profit from the marketing process; therefore, customers may gain value from the resulting benefits [7]. Finally, consumers anticipate long-term connections such as customer loyalty or repurchase behavior [8]. Because this study focuses on the relationships among perceived mental benefits (PMB), hedonic value (HV), and electronic loyalty (ELOY) in the setting of e-commerce, the SOR model is used as the overarching framework.

This data piece's research constructs and measurement items were developed from previous research (Table 1). Three constructs are used in this study, i.e., perceived mental benefits measured by four items [9], the hedonic value measured by four items [10], and electronic loyalty measured by three items [11,12]. These measurement items were adopted and adjusted in expert interviews and group discussions. Further, the data set also included respondents’ demographics as gender, occupation, and frequency of online purchase in one month.

**Table 1:** Scale measurement.

Construct	Items	Code
Perceived Mental Benefits	I have got the perceived enjoyment as I buy the product/service from the e-commerce website	PMB1
	I have got the perceived social interaction as I buy the product/service from the e-commerce website	PMB2
	I have got the perceived discreet shopping as I buy the product/service from the e-commerce website	PMB3
	I have got the perceived control as I buy the product/service from the e-commerce website	PMB4

Hedonic value	Compared to what I spend, I feel online shopping is a pleasure	HV1
	Compared to what I spend, I feel online shopping is happy	HV2
	Compared to what I spend, I feel online shopping is entertaining	HV3
	Compared to what I spend, I feel online shopping is comfortable	HV4

Electronic Loyalty	I expressed the preference with this e-commerce website; for example, I will make a website the first choice or mention this website to my friends	ELOY1
	I enjoyed the patronage of this e-commerce website; for example, I make interactions, like, sharing the news on this website	ELOY2
	I pay the premium for this e-commerce website; for example, I will disclose my personal information to this website	ELOY3

The five-pointed Likert scale was used to assess the PMB, ELOY, and HV scales (one is total disagree; five is total agree). There were two significant reasons why this research utilized a five-point Likert scale rather than a seven-point scale. First, this is an online survey; thus, respondents respond promptly, even on the bus or at work. The second reason is the popularity of the five-point Likert scale in Vietnamese. The questionnaire and study data are accessible in Mendeley data at https://data.mendeley.com/datasets/n9tdpdp45k. This data set includes

**Fig. 1:**
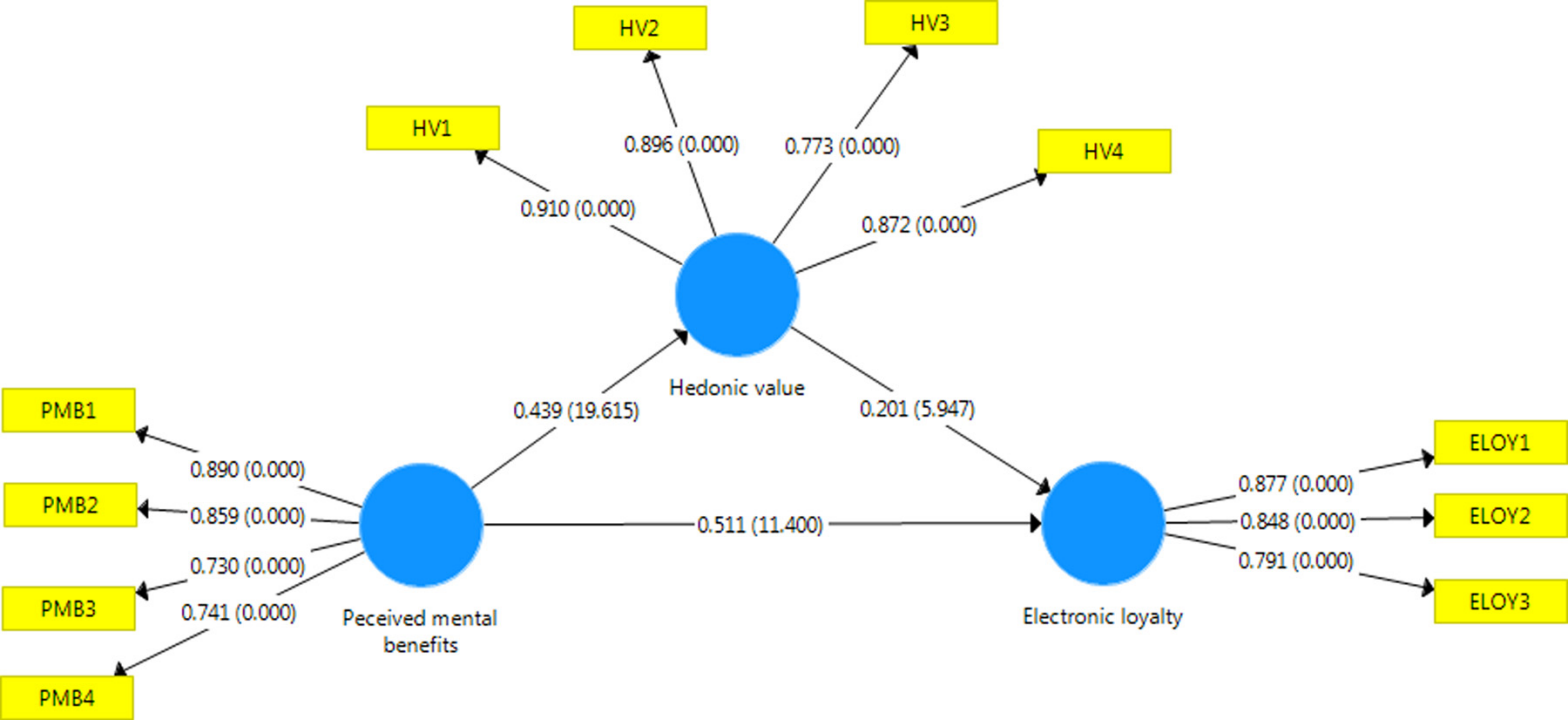
A bootstrapping result model.

• a data file (data.csv) containing 485 records and three research factors: electronic loyalty (three items), perceived metal benefits (four items), and hedonic value (four items);
• a questionnaire (Questionnaire.pdf), which describes the data-collection tool in this study; and
• a figure file (Fig. 1. png), which shows the bootstrapping result in SmartPLS 3.7 software.

## 2 Experimental Design, Materials and Methods

Before collecting the data process, a qualitative research method was conducted to confirm the research constructs and adjust the research items. This stage enhances the validity of the study's measurement scales [13]. To gather information for the analysis, field research techniques were utilized as well as methods for expert interviews and group discussions to collect data for qualitative approaches, in which Group 1 consists of eight e-commerce professionals for in-depth interviews; Group 2 consists of 16 MBA students who often buy online for focus group discussions.

A survey was performed in the three most prominent cities in Vietnam: Ha Noi, Da Nang, and Ho Chi Minh, with the highest e-commerce [14]. Data for quantitative analysis are gathered via a self-administrated questionnaire to customers who frequently purchase online, such as white-collar employees, business owners, lecturers, housewives, workers, and government officials. The convenience sampling technique was conducted because these data were collected via Internet-based survey. Most Web surveys have been done on convenience samples with a readily available list of target demographics as e-commerce customers [15]. Moreover, due to the online questionnaire being distributed on social media platforms, the convenience sampling method will increase response rates, timeliness, and reduce cost [15,16]. The online questionnaire was created using Google Forms and disseminated through social media platforms such as Facebook and Zalo, two popular social network media in Vietnam. The sample size for this research was 485 participants, including 246 men (50.7%) and 239 women (49.3%); further, 14.6% is the percentage of each occupation as a student, lecturer, government official; 72 participants are workers, accounting for 14.8%; the portion of the white-collar employees, business owners, housewives are, respectively, 14.2%, 13.4%, 13.6%. All respondents purchase from e-commerce websites two times a month, in which 27% of them shop more than 10 times Table 2. depicts the demographics and characteristics of the participants in this survey.

**Table 2:** Descriptive demographic statistic.

		Frequency (n)	Percentage (%)
Gender	Male	246	50.7
	Female	239	49.3

Occupation	Students	71	14.6
	White-collar employees	69	14.2
	Business owners	65	13.4
	Lecturers	71	14.6
	Workers	72	14.8
	Housewives	66	13.6
	Government officials	71	14.6

Frequency of online shopping per month	2 - 4 times	116	23.9
	5 - 6 times	116	23.9
	7 - 10 times	122	25.2
	More than 10 times	131	27.0

The data set was processed as the analytical method developed by Hair et al. [17], including (1) assessing the scale's reliability and validity, (2) assessing multicollinearity, analyzing the path coefficient, and assessing indicators of R^2^, f^2^, and Q^2^. Finally, the research evaluated the mediating role of hedonic value in the conceptual model.

### 2.1 Stage 1. Reliability and validity assessment

A Cronbach's alpha coefficient (CA) higher than 0.7 was used to evaluate scale reliability. A scale has convergent validity when the composite reliability (CR) is equal to or more than 0.7, the average variance extracted (AVE) value is equal to or larger than 0.5, and the outer loadings of the items in constructs (OL) are similar to or greater than 0.708. Moreover, discriminant validity is assessed through the heterotrait–monotrait ratio of correlations (HTMT), which is less than 0.85. Following the data analysis result in Table 3, this study's measurement scales demonstrate reliability and validity.

**Table 3:** Reliability and validity assessment.

					HTMT	
	CA	CR	AVE	OL	ELOY	HV
ELOY	0.792	0.877	0.705	[0.791 - 0.877]		
HV	0.893	0.922	0.747	[0.773 - 0.910]	0.456	
PMB	0.83	0.882	0.653	[0.730 - 0.890]	0.643	0.428

### 2.2 Stage 2. PLS-SEM assessment

First, data were processed to evaluate the criteria proposed by et al. [17] as the coefficient of determination (R^2^), effect size (f^2^), predictive relevance (Q^2^), and variance inflation factor (VIF). R^2^ of 0.2 is regarded as high in social research [17]; consequently, the R^2^_HV_ was 0.193, indicating a poor degree of interpretation of PMB to HV, although this result may be acceptable. PMB and HV explained a modest ELOY change with R2ELOY in the study model with 0.392. The effect level is slight, moderate, and high when f^2^ is 0.02, 0.15, or 0.35. According to Table 4, the impact size of PMB on ELOY and HV was the moderator. Q^2^_ELOY_ and Q^2^_HV_ are more significant than 0; hence, the predictive significance of the route model for a given dependent construct is shown by a specific reflecting endogenous latent variable. Last, data analysis pointed out no co-linearity in the model, as all VIF are smaller than 3.

**Table 4:** R^2^, f^2^, Q^2^, VIF value.

		f^2^			VIF	
	R^2^	ELOY	HV	Q^2^	ELOY	HV
ELOY	0.392			0.262		
HV	0.193	0.054		0.117	1.239	
PMB		0.348	0.239		1.239	1

The partial least-squares structural equation modeling (PLS-SEM) was used to assess the relationship between variables. In Fig. 1, the PLS-SEM shows that all relationships in the research model are supported. The regression weights provided in Fig. 1 are used to assess the relationship between statistical variables in the PLS-SEM model. The PLS-SEM shows that all relationships in the research model are supported. According to PLS-SEM analysis, perceived mental benefits have a positive impact on electronic loyalty (beta = 0.511 > 0; *t*-value = 11.4 > 2.58) and hedonic value (beta = 0.439 > 0; *t*-value = 19.615 > 2.58). Moreover, the hedonic value had a positive effect on electronic loyalty (beta = 0.201 > 0; *t*-value = 5.974 > 2.58). Hence, PLS-SEM data analysis proved that all relationships in the research model are supported at 99% of the confidence level.

### 2.3 Stage 3: mediator assessment

The function of hedonic value (HV), as a mediator in the connection between perceived mental benefits (PMB) and electronic loyalty (ELOY), is evaluated using four criteria [18]:

• Criterion (1): PMB significantly impacts HV
• Criterion (2): HV significantly impacts ELOY
• Criterion (3): PMB significantly impacts ELOY
• Criterion (4): The impact of PMB on ELOY is not significant or decrease under the effect of HV

After analyzing the survey data in SmartPLS software, the result in Table 5 shows that all criteria to assess the mediating role of hedonic value are satisfied. Therefore, hedonic value is a partial mediator in relationship-perceived mental benefits and electronic loyalty.

**Table 5:** Mediating effect assessment.

	Original Sample	Standard Deviation	T Statistics	Result
HV -> ELOY	0.201	0.034	5.947	Supported
PMB -> ELOY	0.511	0.045	11.4	Supported
PMB -> HV	0.439	0.022	19.615	Supported
PMB -> HV -> ELOY	0.088	0.015	5.833	Supported

## Ethics Statement

Ethical approval has been obtained from the Industrial University of Ho Chi Minh city; the real protocol number is 153/2021. Informed consent was obtained from all participants in this study. Participants were free to leave the survey at any time. I respect participants’ privacy rights as ethical research. As a result, the data provided do not identify individuals based on their answers. The poll was entirely anonymous, with no information that could be used to determine participants’ identities.

## CRediT authorship contribution statement

**Bui Thanh Khoa:** Conceptualization, Methodology, Data curation, Investigation, Writing – original draft, Software, Validation, Writing – review & editing.

## Declaration of Competing Interest

The authors declare that they have no known competing financial interests or personal relationships that could have influenced the work reported in this paper.

## Appendix Supplementary materials

Image, application 1
