# Supplementary material for: Dataset for the electronic customer relationship management based on S-O-R model in electronic commerce
Source: Data Brief. 2022 Mar 12;42:108039. doi: 10.1016/j.dib.2022.108039 (PMC8933815; doi:10.1016/j.dib.2022.108039)
Supplement: Supplementary file 1 [file mmc1.pdf]

# QUESTIONNAIRE

Dear  Sir/Madam!

My name is Bui Thanh Khoa; I am a researcher at the Industrial University of Ho Chi Minh city

I am working on the “Dataset for the electronic customer relationship management based on S-O-R model in electronic commerce” paper. The success of the project depends on your answers to this survey. The information you provide will be kept confidential and used only for academic research.

If you have any questions, please contact us at email buithanhkhoa@iuh.edu.vn

Thank you very much for your interest and time in participating in this survey.

## I. Screening questions

*1. Have you ever purchased goods on e-commerce websites (online purchases)?*

☐ Have Bought

☐ Never bought (stop survey)

*2. When was the last time you made an online purchase?*

☐ Less than one week

☐ One week ago

☐ One month ago

☐ Three months ago.

☐ Six months ago (stop survey)

☐ One year ago (stop survey)

☐ More than one year (survey stop)

## II. Main questions

Please indicate your opinion by circling (or marking an X) the number that best describes your level of agreement with each of the following statements: 1 = Totally disagree; 2 = Disagree; 3 = Neutral; 4 = Agree; 5 = Totally agree.

| **Perceived Mental Benefits** | | | | | |
|---|---|---|---|---|---|
| I have got the perceived enjoyment as I buy the product/service from the e-commerce website | 1 | 2 | 3 | 4 | 5 |

| | | | | | |
|---|---|---|---|---|---|
| I have got the perceived social interaction as I buy the product/service from the e-commerce website | 1 | 2 | 3 | 4 | 5 |
| I have got the perceived discreet shopping as I buy the product/service from the e-commerce website | 1 | 2 | 3 | 4 | 5 |
| I have got the perceived control as I buy the product/service from the e-commerce website | 1 | 2 | 3 | 4 | 5 |
| **Hedonic value** | | | | | |
| Compared to what I spend, I feel online shopping is a pleasure | 1 | 2 | 3 | 4 | 5 |
| Compared to what I spend, I feel online shopping is happy | 1 | 2 | 3 | 4 | 5 |
| Compared to what I spend, I feel online shopping is entertaining | 1 | 2 | 3 | 4 | 5 |
| Compared to what I spend, I feel online shopping is comfortable | 1 | 2 | 3 | 4 | 5 |
| **Electronic Loyalty** | | | | | |
| I expressed the preference with this e-commerce website; for example, I will make a website the first choice or mention this website to my friends | 1 | 2 | 3 | 4 | 5 |
| I enjoyed the patronage of this e-commerce website; for example, I make interactions, like, sharing the news on this website | 1 | 2 | 3 | 4 | 5 |
| I pay the premium for this e-commerce website; for example, I will disclose my personal information to this website | 1 | 2 | 3 | 4 | 5 |

## III. Demographic questions

Please indicate your opinion by circling (or marking an X) the number that best describes your demographic

| | | |
|---|---|---|
| **Gender** | Male | 1 |
| | Female | 2 |
| **Occupation** | Student | 1 |
| | White-collar employee | 2 |
| | Business owner | 3 |
| | Lecturer | 4 |
| | Worker | 5 |
| | Housewife | 6 |
| | Government official | 7 |
| **Frequency of online shopping per month** | 2 - 4 times | 1 |
| | 5 - 6 times | 2 |
| | 7 - 10 times | 3 |
| | More than ten times | 4 |
